# Experience with single transscrotal orchidopexy for palpable cryptorchidism in Vietnamese children

**DOI:** 10.1038/s41598-025-94261-1

**Published:** 2025-03-27

**Authors:** Thach N. Pham, Lai V. Chu, Truc T. Nguyen, Yen L. Nguyen, Linh U. N. Truong, Martin T. Corbally, Ho T. Ban

**Affiliations:** 1 Department of Pediatric Surgery, Children’s Hospital 2, 14 Ly Tu Trong, Ben Nghe Ward, District 1, Ho Chi Minh City, Vietnam; 2 Department of Pediatric Surgery, University of Medicine and Pharmacy at Ho Chi Minh City, Ho Chi Minh City, Vietnam; 3 Department of Surgery, Royal College of Surgeons in Ireland – Medical University of Bahrain, Busatein, Bahrain

**Keywords:** Cryptorchidism, Scrotum, Orchidopexy, Medical research, Urology

## Abstract

This study aims to assess the effectiveness and outcomes of a single transscrotal incision orchidopexy for palpable low-lying undescended testes. A prospective cohort study was conducted using a single transscrotal incision for all cases. Intraoperative clinical variables and outcomes were evaluated. Between October 2022 and February 2023, 47 patients at Children’s Hospital 2, Ho Chi Minh City, underwent 53 orchidopexies using a single transscrotal incision. The median operative time was 25 min (range: 20–40 min). Mild scrotal edema occurred in seven patients, with no major postoperative complications. A patent processus vaginalis was detected intraoperatively in 50 cases. Fifty-two of 53 testes (98%) were positioned successfully in the scrotum, with one testis slightly higher but deemed satisfactory. The mean testicular volume increased significantly from 224.6 mm^3^ preoperatively to 301 mm^3^ postoperatively (p < 0.05). The surgical success rate was 98%, with all patients satisfied with the cosmetic outcome. No cases of hernia or hydrocele were observed postoperatively. Single transscrotal incision orchidopexy is a safe and effective technique for palpable low-lying undescended testes and should be considered as a viable alternative approach.

## Introduction

Cryptorchidism (undescended testes) is a common congenital anomaly with an incidence of 1–2% at 3 months. Of these 80–90% are palpable and 10–20% non-palpable. Palpable cryptorchidism is typically approached via a two incision orchiopexy with successful placement in the scrotum in 89 – 100% of patients^1^. Bianchi^2^ introduced the single-incision transscrotal orchidopexy in 1989; however, this approach has not yet gained widespread acceptance. The single incision orchidopexy carries many potential advantages such as no disruption of groin anatomy, less post-operative pain and a faster operative time^2–4^.

This technique has not been widely adopted, primarily due to concerns regarding access for hernia sac excision, insufficient length of the spermatic cord, and adherence to traditional, well-established techniques. Therefore, we conducted this study^5,6^. The objective of this study is to evaluate the short-term outcomes of this method in the treatment of palpable low undescended testes in children.

## Materials & methods

This prospective cohort study was carried out between October 2022 and February 2023 at the Dept. of Surgery, Children’s Hospital 2, Ho Chi Minh City, Vietnam. Patients attending the day ward for elective orchidopexy were included.

### Inclusion criteria

All patients were under 16 years and were fully consented to use this approach as a new departure from what had been standard surgery at our hospital.

The primary outcome of the study was the surgical success rate, while secondary outcomes included operative time and patient satisfaction.

Patients were diagnosed with palpable low-lying cryptorchidism if they met the following criteria:

+ The patient had no testis in the scrotum since birth.

+ The testis was palpable and located along the pathway from the inguinal canal to the scrotum.

+ When the spermatic cord was stretched, the lowest position of the testis reached the scrotum.

+ Upon release of the stretched spermatic cord, the testis retracted above the scrotum immediately.

Inguinal cryptorchidism was diagnosed when the testis was located above the pubic tubercle, while ectopic testis was diagnosed when the testis was located below the pubic tubercle.

Clinical inguinal hernia was defined based on observations by the parents and/or the surgeon, characterized by a painless, reducible groin swelling.

Patent processus vaginalis (PPV) was confirmed when probing with forceps or a Kelly clamp revealed communication between the tunica vaginalis and the peritoneal cavity.

Ligation of the processus vaginalis was performed at the level of the deep inguinal ring, close to the preperitoneal fat or the inferior epigastric artery.

Testicular volume measurement: The volume of all testes was measured by ultrasound using the formula:

Testicular Volume = Length (L) × Width (W)^2^ × 0.52. This formula was applied according to the study by Tseng^7^.

Final parental satisfaction outcome was assessed at 6 months post-operation using a simple questionnaire. Parents rated their satisfaction based on their subjective perception of the surgical scar visibility with the options: “very satisfied,” “satisfied,” or “not satisfied.”

### Exclusion criteria

Any patient with a history of previous inguinal or scrotal surgery, congenital anomaly such as bifid scrotum, penoscrotal transposition, or proximal hypospadias, high-positioned testes or if the patient was lost to follow up.

Testicular volume was measured by ultrasound prior to surgery and again at 6 months follow up. The Wilcoxon rank Test was used to compare volumes and considered significant when the volume had decreased by 50% in the post-operative period. We used SPSS software version 2000 for the data analysis.

Potential biases include single-center recruitment bias and measurement bias associated with operator-dependent ultrasound assessments.

### Brief description of the technique

**Fig. 1:**
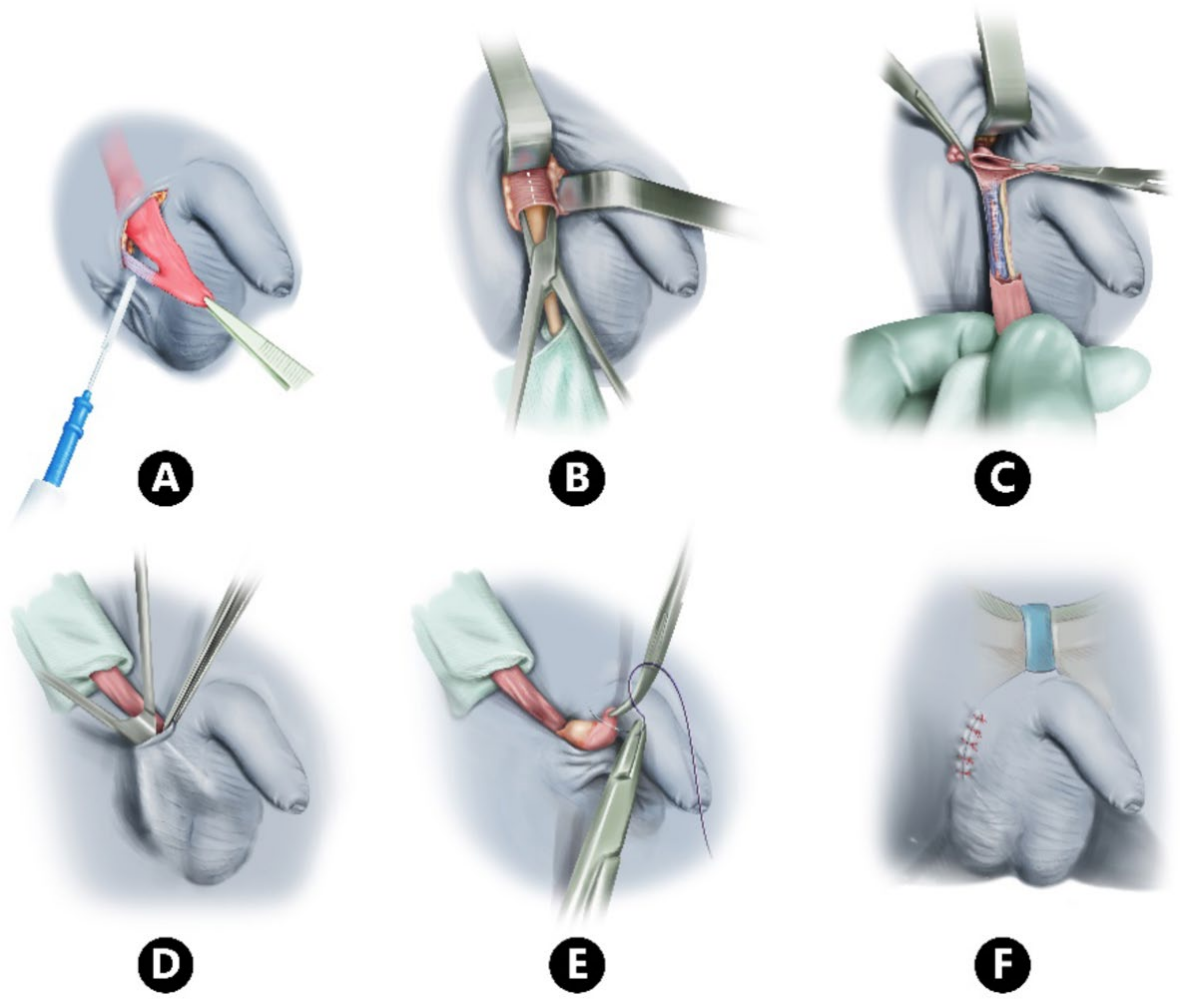
Steps in single transscrotal incision orchidopexy.

An incision 1.5–2 cm in the uppermost fold between the scrotum and groin region is made toward the external ring, allow to secure the external spermatic pouch, the gubernaculum dissection continues to mobilize the pouch and resect all the restriction bands (Fig. 1A). A small proximal retractor was used to provide more exposure and ligate the processus vaginalis (if present). Occasionally a small incision was needed in the ext. oblique at the deep ring/inguinal canal (0.5–1 cm on the front wall of inguinal canal) especially with testes that retracted back to this area (Fig. 1B, C). All the dissection to get the competent length for tension free placement of the testes within the scrotum is shown in (Fig. 1D, E). We create a dartos pouch by using two Kelly clamps to grasp the dartos layer at the lower edge of the incision. Then, scissors are used to separate the skin from the dartos layer, forming the pouch. The testis is brought down into the scrotum by passing it through the dartos layer and securing it with a Vicryl suture between the lower pole of the testis and the base of the pouch (Fig. 1F).

All patients were performed in the day surgery department by only one team, and were followed up at 1 week, 1, 3, and 6 months. All positions of the testis after orchidopexy were defined: right desired position (low or middle of the scrotum), not good (upper half scrotum or outside scrotum after 6 months follow-up).

The volume of all testes were measured by ultrasound^7^. Testicular atrophy was judged to have occurred if the volume was less than half the pre-operation size.

The final parental satisfaction outcome was graded at 6 months post-operation as follows: very pleased, pleased and unhappy. Surgery was considered a success when there were no complications, the testis was in an acceptable scrotal position and there was no measurable atrophy at 6 months follow-up.

**Fig. 2:**
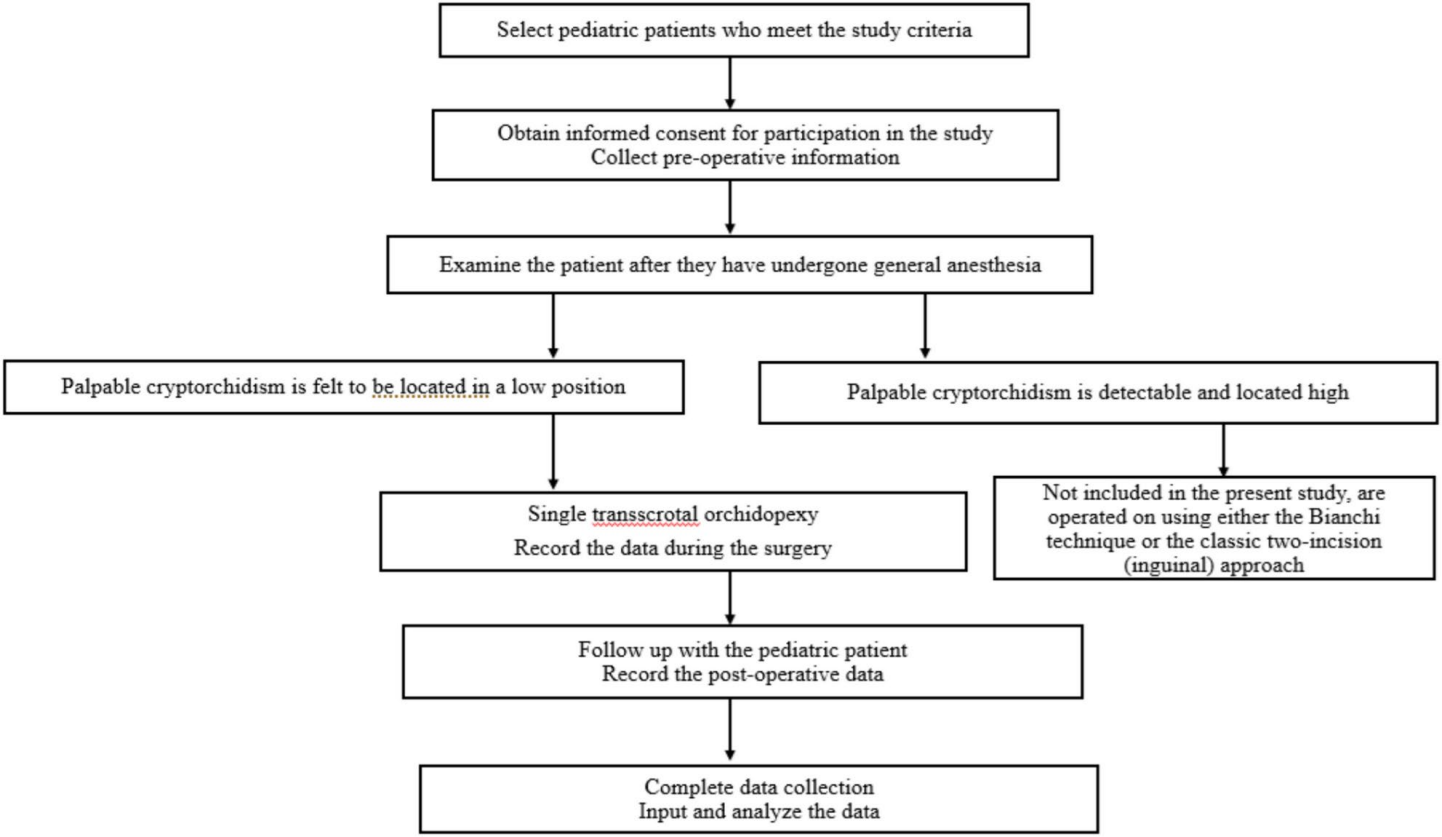
Research flow diagram.

The research process is illustrated in Fig. 2.

This study was approved by the Ethics Committee of Children’s Hospital 2, Vietnam, under approval number 813/GCN-BVND2. All experiments involving human subjects were conducted in accordance with relevant guidelines and regulations.

Informed consent is obtained from the parent and/or legal guardian for study participation.

## Results

Fifty three palpable low-lying cryptorchidism from 47 patients were eligible for our study, 22 cases on the right side, 19 cases on the left side, and six bilateral. Median age was 26.6 months (9–147 months). The testes were found to be in the inguinal canal in 12 cases, outside the inguinal canal in 41 cases, without clinical hernia symptoms in 43 cases, and with an obvious clinical hernia in 10 (Table 1).

**Table 1:** Clinical features of the patients.

Features	Value
Age (median)	26.6 (9–147) months
≤ 36 months	32 (68%)
37–60 months	8 (17%)
≥ 60 months	7 (15%)
Cryptorchidism side (%))	
Right side	22 (46.8%)
Left side	19 (40.4%)
Bilateral	6 (12.8%)
Original position of the testis (n (%))	
Canalicular	12 (22.6%)
Outside inguinal canal	41 (77.4%)
Symptoms of hernia on investigations before surgery (n (%))	
No symptom	43 (81%)
With symptoms of inguinal hernia	10 (19%)
Testicular volume pre-operation (mm^3^)	
Affected testis (median)	215.3 (76.5–590.7)
Descended testis (mean ± SD)	393 ± 126.7
Patent processus vaginalis (n (%))	
Yes	50 (94.3%)
No	3 (5.7%)
Ligation of processus vaginalis (n (%))	
At the deep inguinal ring	44 (88%)
As high as possible	6 (12%)
Duration of surgery median (range)	25 (20–40) min

This table presents the demographic and clinical characteristics of the patients in the study. Variables include age distribution, laterality of undescended testis (UDT), original testicular position, presence of inguinal hernia symptoms before surgery, preoperative testicular volume, and the presence of a patent processus vaginalis. Data are expressed as median (range) for continuous variables and as number (percentage) for categorical variables.

The median operation time was 25 min (20–40 min), a patent processus vaginalis was found in 50 cases, but three had no processus vaginalis (5.7%). All patent processus vaginalis were ligated as high as possible or close to the deep inguinal ring.

The median follow-up time was 7 months (5–7 months). No patients sustained a wound infection but seven experienced mild scrotal edema which resolved after 1–2 weeks. An excellent scrotal position was obtained in 52 patients but in one the testis was a little high but still acceptable.

Cosmetic satisfaction gained in 100%, 52 cases (98%) very pleased with the wound, many cases seemed invisible. No patients experienced surgical site infections; however, seven cases developed mild scrotal edema, which resolved spontaneously within 1–2 weeks without any intervention.

Testicular volume was measured 6 months after surgery. An increase in testicular volume was observed after orchidopexy, with a mean difference of 78.5 mm3 between preoperative and postoperative measurements (95% confidence interval: 10.9–146.2). The difference is statistically significant with p < 0.05 (Table 2). No case testicular volume decreased below 50% compared to pre-operation. The success of this surgery was 98%.

**Table 2:** Testicular volume pre-operation & post-operation.

Variety	Volume (mm^3^)	p*
	Median	
Pre-operation	224.6 (76.5–660.8)	< 0.05
Post-operation	301 (104–2446.1)	

This table presents the changes in testicular volume before and after surgery. The preoperative and postoperative testicular volumes are reported as the median (range). The statistical significance of the difference between preoperative and postoperative testicular volumes was assessed using the Wilcoxon test.

A p-value of < 0.05 indicates a statistically significant increase in testicular volume after surgery.

*Wilcoxon test.

## Discussion

Our study’s age is rather higher than normal, with 32% of patients older than 3 years. It has been suggested that orchidopexy in older children is less successful and that orchidopexy should be performed before the age of 2 years^4,8,9^. We had no difficulties in doing this technique in older patients, an experience shared by others^6,10^. While it may be controversial it appears that the age of surgery does not affect the surgical outcome, but older age may affect function. We might need to look at fertility and spermatogenesis above 1 or 2 years here. Our patients are older because of social and cultural factors specific to Vietnam, and this demographic may take some time to change. Talabi^11^ insisted the age at the time of surgery is not the major factor to affect the outcome of the surgery.

Some authors take this single transscrotal incision orchidopexy as the preferable approach in obese patients avoiding longer inguinal incisions^12^. We believe this to be an important consideration as in obese boys the dissection required is greater and more difficult and the risk of iatrogenic torsion is higher in a two-incision approach^13^. Choosing a high scrotal skin incision and creating a dartos pouch after ligation and division of the processus vaginalis helps minimize surgical field obstruction by adipose tissue.

In our 53 cases, 22.6% testes were in the inguinal canal, but this did not limit the ultimate result of a scrotal testis in 52 of 53 procedures. In our study we noted that when the testis can be brought close to the scrotal neck (under general anesthesia) that a scrotal approach is a viable option^14–16^. We think that when the testis is palpable and can be milked out of the ring, then it is suitable for a scrotal approach.

Misra^17^advised that the high scrotal orchidopexy approach should not be applied in cases in which there is an obvious patent processus vaginalis. We don’t agree with this opinion and the presence of a hernia or an obvious patent processus is not a contraindication to this approach as 43 of our patients had this anatomy with no impact on the approach or the outcome^12,17^. We believe that the sac or PPV can be ligated at the normal position in the majority of children via a single scrotal incision. There is no statistical difference in surgical time between the group with patent processus vaginalis and without it, similar to Takahashi^18^. We believe that proactive division of the anterior wall of the inguinal canal in all cases is key to successful ligation of the processus vaginalis, especially in cases with concurrent inguinal hernia.

Although not within the scope of our study, the scrotal incision may enhance cosmetic outcomes when used for inguinal hernia repair in children, particularly in cases of bilateral hernias. However, it may increase the risk of surgical site infection and does not demonstrate a significant difference in operative time compared to the traditional inguinal approach^19^. Additionally, the recurrence rate of inguinal hernia is 0.67%, and there have been reports of acquired undescended testes following surgery^20^. We believe that inguinal hernia repair via the scrotal approach is feasible; however, selecting the appropriate incision (median raphe, mid-scrotal, or inguinoscrotal) and proactively dividing the anterior wall of the inguinal canal for high ligation of the processus vaginalis is crucial to minimize postoperative hernia recurrence.

Testicular size may increase or decrease postoperatively^21,22^, but it generally shows a significant increase compared to preoperative measurements after an average follow-up of 2.5 years^7^. Therefore, the short postoperative follow-up period may explain the cases of decreased testicular size in our study. Moreover, aside from operator dependency, ultrasound image quality obtained in the inguinal region may differ from that of the scrotal region. Additionally, our testicular volume measurement relied only on the length and width dimensions from ultrasound imaging, which may introduce measurement errors.

Although subjective and focused solely on aesthetics, all families were satisfied with the appearance of the surgical scar. The aesthetic aspect of the scrotal incision is considered one of the advantages of the procedure, providing high cosmetic value and reducing potential psychological distress for the patient^2^.

While various factors are cited that explain the outcome of successful orchidopexy (scrotal position with no volume decrease) it is possible that mobility of the testis while under general anesthesia is the most predictable variable and as such the approach (one or two incisions) may not impact on outcome^11,16^. In this regard we have shown an excellent outcome (98% success) via a single incision with a satisfactory position, no loss of testicular volume and a desirable cosmetic result.

We saw no significant complications apart from mild scrotal edema and none of our patients required a second incision. This supports previous reports on the success of the single incision (Table 3).

**Table 3:** Review of the published literature regarding single transscrotal incision orchidopexy.

Authors, year	No. of cases	Type of cryptorchidism	Conversion to two-incision procedure (%)	Follow-up outcome			
				Inguinal hernia (%)	Testicular atrophy (%)	Testiscular ascension (%)	Cosmetic satisfaction (%)
Bianchi^2^	120	Palpable	4.2	0	0	0	NR
Russinko^15^	85	Palpable low-lying	1.2	0	0	1.2	NR
Rajimwale^9^	100	Palpable	6	0	0	0	NR
Bassel^14^	121	Palpable low-lying	0	0	0	0	NR
Samuel^6^	206	Palpable low-lying	0.5	0	0	0	100
Al-Mandil^10^	63	Palpable	0	3	0	1.6	NR
Takahashi^18^	49	Palpable low-lying	0	0	0	2.3	NR
Yecel^16^	74	Mobile (palpable low-lying)	0	0	0	0	NR
	14	Immobile (high palpable)	57	0	0	0	
Arena^13^	205	Palpable low-lying	3.9	0.5	0	0	NR
Talabi^11^	31	Palpable	0	0	3.2	9.6	100
Badbarin^8^	31	Palpable	0	0	0	6.7	100
Elekiabi^4^	20	Palpable	0	0	0	0	100
Our study	53	Palpable low-lying	0	0	0	2	100

This table summarizes previous studies on single scrotal incision orchidopexy, including the number of cases, type of cryptorchidism (palpable or non-palpable, low-lying, or high palpable), and surgical outcomes. The conversion rate to a two-incision procedure, incidence of inguinal hernia, testicular atrophy, testicular ascension, and cosmetic satisfaction rates are reported. Data are presented as percentages (%). “NR” indicates that the data were not reported in the respective study.

*NR* not report.

Our study is limited by relatively small numbers but is important as it introduces a new concept and procedure in Vietnam.

### Our study has several limitations

The study does not represent all cases of palpable cryptorchidism. It does not answer the question of what percentage of cases of palpable cryptorchidism can be treated with single transscrotal orchidopexy. Due to the non-comparative design, the study does not provide a conclusion regarding the efficacy of single transscrotal orchidopexy in comparison to other techniques. The follow-up period was too short to allow for a conclusive assessment of the long-term effects of single transscrotal orchidopexy on patients who underwent the procedure.

## Conclusions

We believe that single transscrotal incision orchidopexy is a beneficial technique for treating palpable testes that can be well milked into the junction of the scrotum and groin under general anesthesia. This approach offers clinical advantages, including shorter operative time and reduced postoperative discomfort. However, we recognize that our sample size is relatively small, which may limit the generalizability of our findings. Further research with larger sample sizes and long-term follow-up is needed to confirm these results and strengthen their applicability in broader clinical practice. Our prospective study has demonstrated excellent postoperative testicular position, no decrease in volume, and satisfactory cosmetic outcomes.

## Supplementary Information

Supplementary Information.

## Data Availability

Data is provided within the manuscript or supplementary information files.
